# Medico-Chirurgical Transactions, (Vol. XVIII. Part I)

**Published:** 1834-01-01

**Authors:** 


### Medico-Chirurgical Review,

### No. XXXIX.

OCTOBER 1, 1833, to JANUARY 1, 1834.

### I.

### Medico-chirurgical Transactions. Vol XVIII. Part 1. November, 1833, pp. 300—3 Plates.

THE eighteenth volume of this Society's Transactions promises to be a very respectable one, if we may judge by the first half. The communications are thirteen in number, and the contributors are all favorably known to the profession—several of them high on the rolls of fame in the medical world. When we mention the names of Bright, Elliotson, Stanley, Key, Lloyd, Arnott, Hawkins, Langstaff, &c. we are sure that observations from such sources must command the attention of our readers. Without further preface, therefore, we shall proceed to embody a full account of some of the contributions to this volume in the present number of our Journal.

### I. Cases and Observations connected with Disease of the Pancreas and Duodenum. By *Richard Bright*, M.D.

The object of this paper is to draw the attention of the profession to a particular symptom in disease, hitherto but little noticed. Independent of this, however, the distinguished author of the paper hopes that the cases narrated will be found interesting, independently of the subject for which they are introduced.

"The symptom to which I refer is a peculiar condition of the *alvine evacuation*, a portion more or less considerable assuming the character of an *oily* substance resembling fat, which either passes separately from the bowels, or soon divides itself from the general mass, and lies upon the surface, sometimes forming a thick crust, particularly about the edges of the vessel, if the fæces are of a semi-fluid consistence, sometimes floating like globules of tallow which have been melted and become cold, and sometimes assuming the form of a thin fatty pellicle over the whole, or over the fluid parts, in which the more solid figured fæces are deposited." 2.

This oily matter has generally a slight yellow tinge, and very fetid odour. Dr. Bostock, on examination of this substance, declared it to be adipocire. The author has, very wisely, attached little importance to the chemical character of the substance under consideration.

*Case* 1. A clerk in a waggon-office, 49 years of age, sober and regular, became affected, in March, 1827, with immoderate thirst, large discharge of urine, and the usual symptoms of diabetes. In September, he presented the phenomena of jaundice, for which he was treated in various ways by different practitioners. In December, he came under the care of Dr. B. Babington, the narrator of the case. The urine was evidently diabetic—the thirst and appetite great. He was ordered to live on animal food, with a moderate proportion of greens or lettuces, and to take subcarbonate of soda. The quantity of urine diminished on this plan, and, on the 15th Dec. the jaundice being considerable, blue-pill and taraxacum were prescribed. On the 24th Dec. the skin still continued yellow; but the urine was no longer diabetic, and only six pints in the 24 hours. The liver was found to be hard, and distinctly perceptible three or four inches below the ribs. The gall-bladder was enormously distended. The mouth was affected by mercury. On the 28th, the patient began to pass a quantity of "*yellowish fatty matter, much resembling butter that had been melted, and again solidified.*" This matter followed the fæces. Liquor potassæ was substituted for the carb. sodæ. This, by the 31st, had *apparently* done away with the oily character of the motions. But the debility and emaciation steadily advanced —the character of the evacuations was bad—and on the 8th January, 1828, the fatty motions re-appeared. It is unnecessary to pursue the case—the poor man died, worn out, on the 1st of March.

*Dissection.* We shall only select the chief characters, as much of the detail is unnecessary.

"The abdomen contained rather more than a gallon of dark olive coloured fluid. The gall bladder distended with very dark bile, was seen with its fundus projecting when the parietes were first removed. The liver was of a dark olive colour from the bile with which it was pervaded. The ducts were greatly enlarged. The common duct was large enough to admit the little finger freely when passed from above downwards, and its internal surface presented a honeycomb or reticulated appearance, and terminated by a cul de sac in the diseased substance of the pancreas, and at its shut end a rough white deposit had taken place probably either of fibrin or cholesterine.

*The head of the pancreas* formed with some of the surrounding glands, a hard globular mass, round which the duodenum turned, and to which both it and the pylorus were firmly joined, and in two parts, where the pancreas and duodenum were welded together by the disease, ulcers of a hard and scirrhous character had taken place, penetrating the whole thickness of the intestine; one of them of the size of a shilling, and the other not larger than a silver penny piece. The pancreas was hard and cartilaginous to the touch, and of a bright yellow colour.

A section of the liver looked like a fine grained dark greenstone porphyry, or very dark Aberdeen granite; the ducts, which were throughout enlarged, being completely filled with bile which flowed from the incision. In different parts of the liver a few irregular masses occurred of a firm hard consistence, but shaded off into the substance of the liver, and not bearing the appearance of circumscribed tubera. The stomach was slightly vascular. The spleen natural in structure, but its external surface mottled, with cartilaginous deposit. The intestines were tolerably natural, but somewhat opake, and the internal lining rather pale. The kidneys were to external appearance perfectly healthy; but the tubular parts shewed themselves more plainly than usual, when the kidney was torn open, and in some of the tubes were white specks from a deposit either of fibrin or of calculous matter. The pelvis of the kidney was not vascular, but was tinged with bile. The lining membrane of the bladder was remarkably healthy and free from all vascularity; but its net-like appearance bespoke more than usual action in the muscular coat. The aorta and common iliacs were in many patches diseased with bony deposits surrounded by dark spots, where the internal surface had been destroyed by ulceration or absorption." 12.

*Case* 2. This was a female, aged 50, admitted into Guy's Hospital on the 19th November, 1828. She was intensely jaundiced—greatly emaciated—stools of a clay colour—occasional severe pains in the bowels—urine highly tinged with bile. Three months previously she was seized with violent pain in the abdomen attended with diarrhœa, her food passing undigested. These pains still continued to return at intervals up to the time of her admission. They were relieved by pressure. The skin was now permanently yellow for six weeks. Dr. B. concluded that the phenomena depended on some organic obstruction pressing at once on the gall-ducts and pylorus, without the chance of remedy. Some pil. hydrarg. and decoct. aloes c. were given, and in a few days he observed the stools covered with white round masses larger than peas, which he attributed to a dose of castor oil that had been taken. But the woman assured Dr. B. she had often passed such stools when no castor oil had been taken. On the 5th December she was ordered taraxacum. On the 6th some of the fatty matter was shewn to Dr. Bostock. She lingered till the 16th Feb. of the succeeding year, when she died at Gravesend.

"*Sectio Cadaveris, Feb.* 18, 1829.—Skin generally of a deep yellow colour, varying in parts to greenish brown, not very unlike the dark colour of the Creole. General emaciation, but by no means to the extent sometimes seen; indeed on the abdomen, there was a considerable portion of fat of a deep yellow colour.

The lungs were in a very healthy state, except that they were both bound firmly at the posterior part by very strong adhesive bands, and the whole surface was tinged moderately with bile. Heart healthy, but small and not firm.

On opening the abdomen, the omentum rather loaded with fat. No peritoneal disease or adhesion, except on the superior surface of the liver, which was attached in several parts by long adhesions to the diaphragm.

The cause of pressure on the bile ducts was immediately obvious; for, on placing the hand near the pylorus a hard lump of the size of a common egg was easily felt, and was soon discovered to be the head of the pancreas itself, and not the glands surrounding that part, forming a yellow mass like the boiled udder of a cow, almost cartilaginous. Its texture was uniformly hard and unyielding, and the whole pancreas partook of the same, but in a less degree. The head of the pancreas was firmly and inseparably glued to the duodenum, and the hardness very nearly surrounded that viscus. Laying open the duodenum, its internal surface was uneven and ulcerated, the ulcer having eroded the whole of the coats, and in the portion lying on the head of the pancreas it was of a soft consistence and light yellow colour, communicating with the substance of the tumour here irregularly softened or suppurating to the extent of a small chesnut. In the midst of the ulcer a little nipple-like body was seen projecting on its surface, which proved to be the orifice of the common duct of the gall bladder. This was still pervious as the thick bile could be squeezed out of the gall bladder through it. But it was obvious that this had either lately become pervious by the ulceration of its orifice, or the ulceration of the hard mass in which it was imbedded, or that its situation in the contracted duodenum had acted as a compressing cause; for the gall bladder was distended, containing at least four ounces of thick dark green bile which stained the lining membrane of the deepest colour. The gall bladder, though thus loaded, was not tense, and conveyed the idea of a somewhat flaccid bag, so that I should have pronounced it to have been more distended lately than at the present moment. The disease of the mucous coat of the intestines occupied the outside of the ridge forming the pylorus, which was strongly marked, but was not scirrhous; yet on passing the finger before the pylorus was cut open, the hardened neighbouring structure produced all the effect of a stricture.

The liver was of its natural size, containing several round tubera sprinkled through various parts, from the size of a grain of rice to that of a nutmeg. These were not very numerous, but five or six were seen on the superior surface where they were perfectly circular, and a little depressed in the centre. They were decidedly harder than the surrounding liver, but did not separate freely from it; on the contrary, generally seemed to be shaded off into the surrounding parts and the texture of the liver was discernible in them. The larger tubera were soft and yellow in the centre. The general structure of the liver was healthy, rather soft, and of a dark olive green colour. The biliary ducts were enormously distended, their branches near the margins of the liver were visible on the surface, and they were filled with a fluid watery bile.

The mucous membrane of the stomach was rather spongy, of a reddish tint, and it contained half a pint of brown grumous matter, apparently secreted from its surface. We examined several portions of the mucous membrane both of the small and large intestines, but they presented nothing peculiar except a rather spongy texture, and on some parts a grey colour. The spleen was healthy but soft. The kidneys large and flaccid, tinged with bile throughout, particularly their lining membrane. The large vessels appeared healthy and the lumbar and other glands were not diseased. The bladder contained some yellow urine. The uterus was rather thick and round in its form, the cavity large, and the glands at the mouth of that organ put on the appearance of vesicles at first sight. They were distended with glairy almost gelatinous mucus of a yellow colour, which could with some force be squeezed from their orifices." 20.

It is probable, as Dr. B. says, that the disease in the pancreas was the first in order of succession, and had existed for some time before the other commenced. Latterly the biliary ducts were mechanically obstructed, and as the bile could not be properly discharged from the liver, it is a question how far various diseases of this organ and of other parts might be ascribable to this morbid retention of the hepatic secretion.

*Case* 3. Jane Davis, aged 21, was admitted on the 13th July, 1831, with anasarca of the lower extremities. There was also some fluid in the abdomen—countenance sallow, lips purple, some jaundice of the face. She had lived rather irregularly.

"14th. Her bowels had been copiously opened without medicine. I saw the evacuations, they were abundant, of a pultaceous consistence, very deficient in bile, and most dreadfully fœtid. The chamber pot was completely filled, and on the surface was observed a thin scum like a thin film of grease which collected and coagulated as grease more decidedly upon the side of the vessel. The lower and more fluid parts of the contents of the vessel looked slightly tinged with blood. It was doubted whether some parts were not purulent. Placing the hand on the abdomen, the upper part was distended and rather tender, particularly towards the right side, where an indistinct hardness could be felt, somewhat resembling the liver." 22.

Hyd. cum creta, with Dover's powder was ordered. No material alteration occurred; except loss of flesh, the evacuations continuing copious, fetid, and of a dark clay-colour, with fatty pellicles on the surface. She expired on the night of the 19th of the same month.

"*Dissection.* The whole body was decidedly tinged with bile, some yellow serum escaped from the abdomen when it was opened. The liver was immediately seen considerably enlarged from distension, and of a dark olive green colour. The fundus of the gall-bladder projected, it was distended with bile of a dark green colour. The quantity which it contained could not have been less than four ounces, and the ducts were as large as the little finger till they entered the duodenum where the orifice was very small, and it required considerable force to make the bile pass into the intestines. The whole of the intestines were somewhat distended, and in several places, when viewed externally, their puckered and discoloured appearance plainly indicated that mischief had been going on within, and at one part of the small intestines an actual perforation, large enough to admit the point of the little finger had taken place, but a slight adhesion which gave way in the examination prevented the feculent matter from being effused.

The whole course of the intestines was now laid open, and fungoid excrescences and ulcerations were found distributed at irregular intervals from their commencement at the pylorus, to the termination at the colon. Whether any existed in the rectum, I am not quite certain. These ulcers might be traced throughout their whole progress. They began by small elevations, generally upon the edges of the convolutions of a light yellow or white substance, and those which had arrived at the size of a pea, generally had a depression in the centre as if from partial ulceration. The depressed part was softer than the surrounding edge, and if the tumour was squeezed, a whitish puriform or cerebriform matter issued from pores upon its surface. This, however, was better seen when the whole disc had increased to the size of a sixpence. About this time, or sometimes sooner, the surface lost its light and clean appearance, and became covered, sometimes with a sloughy mass, but more frequently with a dark grumous coat, apparently from blood, which had exuded, and become changed on its surface. The mass was now elevated nearly half an inch, the edges inverted or cup-shaped, and the centre either raised with the loose fungoid slough and blood, or if this had come away, was deeply excavated, going on in its progress to perforate the substance. In two instances, these fungoid ulcerations communicated immediately with large fungoid excrescences, probably glands situate externally to the intestine. One of these was close to the ileo-colic valve, where the external ulcer was black with grumous exudation, and formed the mouth of a cavity which would admit the finger into a mass involving the glands of the mesocolon. Another of the same kind, but less completely opening into the external diseased mass, occurred in a portion of the duodenum.

The mesenteric glands were involved in this disease, and the renal capsules, but more particularly the left, had suffered from the same affection.

The kidneys were healthy. The uterus was healthy, but its appendages had suffered great irritation. One of the fimbriated extremities was completely bound down, and the orifice obliterated, and the ovaries were corrugated and contained vesicles in different states of disease.

In the liver no fungoid disease shewed itself, but its texture was natural, though gorged with bile. The pancreas was most deeply involved in the disease. It formed a hard mass near its head, and then a more healthy portion intervening, another hardened mass was seen near to the spleen, when another small portion remained healthy at its termination, so that it might be said to be occupied by two fungoid tubercles, which involved two thirds of its whole structure. The limits of these diseased masses were not distinctly defined, but they were of a more yellow colour than the rest of the organ, and destroyed the lobular structure of the gland.

The spleen was unusually small.

In the chest the same disease was found affecting the bronchial glands, and in the form of one round fungoid tubercle of the size of a moderate plum, in the apex of one of the lungs; this was imbedded completely in the substance, and was of a yellow white colour." 25.

Dr. B. observes that, when we draw a comparison between the three foregoing cases, a very close analogy, or even identity, will, in many circumstances, be traced. In all of them chronic ailment terminated, sooner or later, in jaundice, accompanied by a great peculiarity in the alvine dejections. In all, there were found obstructed biliary ducts—liver gorged with bile—diseased pancreas—and malignant ulceration of the duodenum. But all these analogies and coincidences being granted, we have still a very insecure basis for a theory of the peculiar discharges from the bowels. Dr. B. is inclined to attribute the said peculiarity of discharge to the malignant ulceration in the duodenum.

The two following cases are introduced by Dr. B. in order to shew cause why he doubted the existence of diseased pancreas, the peculiar discharges being absent.

*Case* 4. This was a coachman who had suffered the usual exposures of his situation, and had, for several months, been wasting and growing pallid, complaining of constant deep-seated pain at the scrobiculus cordis going to the back. Although considerably emaciated at the time, Dr. B. could detect no tumor in any part of the abdomen. There was no jaundice, no sickness, nor any appearance of disease in the lungs. None of the fatty matters could be seen in the evacuations. On dissection, there were found scirrhous tumors in the liver, in Glisson's capsule, and at the small curvature of the stomach. The pancreas was sound.

*Case* 5. This was a man, aged 50, who had suffered deep-seated pain at the scrobic-cordis for several months, together with palpitation of the heart, and abdominal pulsation. He wasted much, lost his colour; but no tumor could be felt. It was evident, however, that the heart was diseased. There were no fatty matters in the evacuations. Dr. B. therefore demurred to the idea that there was any disease of the pancreas. He died in about a month.

*Dissection.* "Very general and old adhesions were found between the pleura costalis and the pleura pulmonalis, some parts of the lung were hepatized from old disease, while other parts were emphysematous, and some recent irritation was observable in the bronchial membrane. The heart adhered very closely to every part of the pericardium, and was enlarged in its substance universally. The mitral valves and the semilunar valves of the aorta were slightly diseased.

The liver contained a good deal of blood, which was distributed irregularly between the acini, so as to give a mottled or nutmeg appearance. The acini were light coloured, a little tinged with bile. The gall bladder was full of bile, but not distended beyond its natural size. The ducts were pervious; but it was with some difficulty we could make the bile pass from the gall-bladder to the duodenum, apparently owing to its tenacious condition. The pancreas was perfectly healthy, nor was there any material derangement in the other abdominal viscera." 30.

Two cases are next introduced from the practice of Dr. Hull, of Montrose, where almost all the circumstances of the disease in the first cases, including the fungoid disease of the pancreas, existed, and yet with the absence of the fatty discharges from the bowels. These cases we must notice summarily.

*Case* 6. An old lady had enjoyed excellent health till within six months. In May 1827, she began to have occasional pains in the back and sides, giddiness, dimness of vision (aged 76), palpitation, constipation. To these symptoms were afterwards added constant uneasiness, weight, and sense of distention in the stomach, with the eructations and distressing palpitations. The pulse and tongue were natural—urine scanty, with pinky deposit—skin dry—bowels costive. She could lie only on the left side.

"About the 26th of December, a considerable change took place in the appearance of the evacuations. The urine became of a much higher colour, and the stools bore a whitish clay-like aspect; her countenance assumed a bilious tinge. The pain of the side was much more acute, and fixed to a particular spot. Nausea also was superadded to her other distressing feelings. The jaundice became gradually more and more confirmed, and towards the middle of April anasarca began to occupy the legs, and effusion took place in the abdomen." 33.

Without any great variation in the symptoms she died worn out on the first of June 1828.

*Dissection.* The gall-bladder was enormously distended. The ductus communis was an inch in diameter, and completely obliterated at its duodenal extremity. The contents were about eight ounces of black fluid, resembling black paint. The liver was healthy, but gorged with bile. The stomach was apparently healthy. The duodenum considerably thickened, and somewhat contracted. The pancreas was enlarged, and formed a mass of schirrhous disease, involving the termination of the ductus communis in the same diseased structure, where it was completely impervious. The other intestines were healthy. They were not permitted to examine the thorax.

*Case* 6. A gentleman aged 35, consulted Dr. Hull, 16th June 1831, being afflicted with abdominal tumors. There were several of these, but the two chief masses were, one above, and the other below the umbilicus.

"The upper mass which did not quite reach to the pit of the stomach, was about the size of the fist, and on its upper part there was an elastic portion, as if it were an intestine distended with air, lying upon the tumour. The lower mass lay below, and a little to the left of the umbilicus, rising towards the margin of the ribs on the left side, but not to be traced as passing under them. This gentleman was able to walk about and transact his affairs as usual, and his complexion was good, but he complained of considerable loss of general strength, and this was increasing. He stated that ten months before, having previously enjoyed good health, he was supposed to labour under inflammation of the liver, for which he was bled, and other remedies were employed which gave him relief, but left him weak, and of this he never completely recovered; but he had not perceived the tumours till three months before the present time." 37.

Dr. H. considered them as connected with the omentum and peritoneum. He heard no more of the patient till April, 1832, when he was informed of his death. He learnt that, in the interval, he had been twice jaundiced—his urine high-coloured—stools like clay or yeast—but no fatty substances had been observed.

*Dissection.* The liver was healthy, with the exception of a scirrhous tuber on the convex surface, and a few smaller ones in the substance of the viscus. The gall-bladder was greatly distended with bile of a thick consistence and green colour. The upper tumor was a hard mass connected with the omentum, and attached also to the stomach. The lower tumor arose from the root of the mesentery, displaced the small intestines—involved the aorta and iliacs, the pancreas, kidneys, &c. The pancreas was almost annihilated by pressure. The cystic duct was distended with bile, and obstructed by pressure, though not completely so. The pancreatic duct was almost completely obstructed. The diseased masses were of a round form and almost cartilaginous hardness. Some of them were slightly creamy in the centre—some cetaceous. They were conglomerate. The mucous membrane of the duodenum was decidedly diseased, though incipiently so. It appeared as if the malignant disease was establishing itself in that structure. The chest was not examined.

The two foregoing cases are not very confirmatory of our author's doctrine; and he deserves great credit for bringing them forward, though somewhat adverse to his views. This is too seldom the practice with those who have an hypothesis to establish. Another case is detailed, and which occurred in Guy's Hospital, presenting almost all the phenomena that were seen in the foregoing cases, but still without the fatty matters in the evacuations. We need not notice this case, after the full account which we have given of the others.

" Taking then a general review of the foregoing cases, and recapitulating some of the foregoing observations, we find three instances only, in which the fatty evacuation existed; but in each of them so many morbid causes were combined, that it is necessary, by comparing them with other known facts, to reduce these causes as far as we are able, and in doing this we may observe—1st, that a great deficiency of the biliary secretion is well known very frequently not to produce the effect: 2dly, we also know that most extensive fungoid and melanotic destruction of the liver without jaundice is unattended by this symptom: 3dly, we know that extensive fungoid disease in the liver, with jaundice, does not produce it: 4thly, we know that the more simple and inflammatory diseases of the liver, which cause jaundice, are not characterized by these evacuations: 5thly, we know that the extensive ulceration of the mucous membrane of the intestines, from diseases of other kinds, are not indicated by a discharge of fatty matter: and 6thly, we have every reason to believe that malignant ulceration frequently exists in various parts of the intestinal canal, from the pylorus to the rectum, but more particularly in these two parts, without this symptom. Thus then we bring the circumstances of the diseased structure, as far as they have hitherto attracted observation, in connection with this symptom, within a narrow limit,—*disease probably malignant of that part of the pancreas, which is near to the duodenum; and ulceration of the duodenum itself.* These are the only two conditions, which can be traced as being peculiar to all the three cases, and there is good reason to believe that in one of the other cases which I have stated, where this symptom was wanting, the ulceration between the duodenum and the pancreas was also wanting; while in the next case a healthy portion of the pancreas intervened between the fungoid disease and the duodenum, and the mucous membrane of the intestine was not ulcerated; and in the last case, the disease was probably rather seated in the absorbent glands than in the pancreas itself. In this last case, however, the duodenum was ulcerated, and the pancreatic duct greatly enlarged from obstruction, and therefore the case almost identical with those in which the fatty discharge had been observed.

I am well aware that deductions of this kind bear too much the appearances of sophistry to be very applicable to our reasonings on the phenomena of disease, and I freely own that they afford but slender conviction even to my own mind. I offer them, therefore, in no other view than as hints to be improved by future observers, and I will not even affect to decide, whence the peculiar fatty matter is derived: whether it is to be considered as a vitiated secretion from natural structures, which must here be chiefly mucous membranes; or as a discharge from the diseased and ulcerated parts; or as the product of defective digestion of alimentary matter, depending on the imperfect supply or irregular admixture of the biliary and pancreatic or other secretions, or on the perverted and impeded action of the duodenum." 52.

The modesty and good sense of the latter part of the above extract must be sufficiently obvious—and indeed they are characteristic of the author and of all his contributions to medical science. We would not, on slight grounds, differ with such a man as Dr. Bright; but as every one is invited to contribute his individual experience for the good of the whole, we venture to say that the phenomena in the fæcal evacuations alluded to by Dr. Bright, are, by no means, *necessarily* connected with any organic disease in any one of the sub-diaphragmatic viscera. His own cases, indeed, as well as his own doubts, will bring most readers to this conclusion. We do not, however, ground our dissent on the author's own admissions, but on our own observations.

From no very limited field of experience, we can safely say that the phenomena in question—the fatty substances in the evacuations, so far from being a rare, are, on the contrary, a very common occurrence or symptom, in disordered states of the alimentary canal. There is scarcely a day in the week, on which these "fatty substances" are not brought to us by patients, in bottles or in cups, as strange phenomena, and not less terrifying than strange—TO THEM. People affected with disordered function of the digestive organs are proverbially attentive to every phenomenon and symptom that bear reference to their own health. Modern opinions and doctrines induce them to pay particular attention to the fæcal evacuations. In addition to a variety of morbid feelings, they perceive that they lose flesh and strength. When, therefore, they see in their motions what even physicians call "fatty substances," they very naturally take the alarm, and conceive that they are discharging the fat of their bodies through channels which ought to convey away only the refuse of their nutriment. No wonder, they observe, that their bodies waste, when their fat is running through them! We see this phenomenon so much more frequently in functional than in organic diseases, that we attach but little importance to it, and only look upon it as an index or proof that considerable irritation exists in the line of the intestinal canal—we mean, of course, in the mucous membrane. We consider the said substance to be merely a morbid or unnatural secretion from the mucous glands—and possibly from other glands of larger size, as the pancreas. We have frequently seen this discharge change its character, and assume the appearance of tough and orange-coloured jelly, that might be drawn out to almost any length, so tenacious were its particles of each other. This appearance, we have little doubt, was dependent on deranged biliary secretion, all the other symptoms being in support of this opinion. These fatty and albuminous discharges so generally disappear under proper diet and medicines, that we had but one opportunity of examining the body after death, where the phenomenon was conspicuous. In this case the liver was enlarged and tuberculated—and the mucous membrane of almost the whole of the intestines was more or less thickened, and affected with chronic inflammation,

We have made it a rule, when patients have mentioned this peculiar discharge, to put them upon rigid, simple, and light diet—with a few grains of hyd. cum creta and pulv. ipecac. compos. at night; and rhubarb and magnesia in the morning. After a short course of this kind, alkaline bitters, with attention to diet and mild aperients, have removed these appearances from the fæces, and restored the abdominal functions to a healthy state.

It is curious that, in the same volume, two other, and lengthy papers on the subject, are published—one from the pen of Mr. Lloyd—the other from that of Dr. Elliotson. We shall notice these in our next number.

